# Comparative Study on Three Different Designs of Locking Mechanisms in Total Knee Arthroplasty

**DOI:** 10.3390/bioengineering12020169

**Published:** 2025-02-10

**Authors:** Byung Woo Cho, Hyoung-Taek Hong, Yong-Gon Koh, Kwan Kyu Park, Kyoung-Tak Kang

**Affiliations:** 1 Department of Orthopedic Surgery, Severance Hospital, Yonsei University College of Medicine, Seoul 03722, Republic of Korea; chobw0704@yuhs.ac; 2 Skyve R&D LAB, Seoul 07217, Republic of Korea; 3 Joint Reconstruction Center, Department of Orthopedic Surgery, Yonsei Sarang Hospital, Seoul 06702, Republic of Korea; 4 Department of Mechanical Engineering, Yonsei University, Seoul 03722, Republic of Korea

**Keywords:** total knee arthroplasty, locking mechanism, disassembly test, assembly test

## Abstract

The locking mechanism of the fixed-bearing tibial insert is a crucial factor in total knee arthroplasty. Previous studies have predominantly been retrieval-based, with no research examining the forces required for disassembly and assembly based on the design of the tibial insert’s locking mechanism. This study aimed to measure the disassembly and assembly forces of three different locking mechanism designs. Group 1 featured a dovetail design, Group 2 had a peripheral rim design, and Group 3 combined a snap-fit mechanism with a dovetail design. Among the groups, Group 1 exhibited the highest disassembly force (379 ± 42 N), followed by Group 3 (342 ± 58) and then Group 2 (269 ± 18). Similarly, Group 1 also demonstrated the highest assembly force (71 ± 3); however, Group 3 showed a lower assembly force (48.7 ± 2.1) compared to Group 2 (49.7 ± 1.5). These results suggest that design modifications can produce mechanisms requiring minimal assembly force while maintaining strong resistance to disassembly. Due to its snap-pit structure, Group 3 exhibited the lowest assembly force while utilizing the dovetail mechanism to demonstrate a strong disassembly force. The rigorous analysis and robust methodology employed in this study ensure the reliability of the findings, which can serve as a reference for future research and development in this field.

## 1. Introduction

Fixed-bearing (FB) total knee arthroplasty (TKA) has gained popularity in recent product designs due to its comparable radiological outcomes, range of motion, pain relief, and patient satisfaction relative to mobile-bearing TKA, as well as a lower incidence of complications such as polyethylene (PE) dislocation [1,2,3].

Secure fixation between the insert and the tibial component is crucial for successful outcomes in FB TKA. Proper fixation minimizes the risk of PE dissociation, reduces backside wear [4,5,6], and enhances implant longevity [7]. In TKA, the baseplate insert locking mechanism is primarily subjected to compressive stress rather than tensile stress [8], making PE dissociation an uncommon complication. However, when it occurs, surgical intervention is required, and multiple case reports in the literature have documented such events [8,9,10,11,12,13,14,15]. The primary causes of PE dissociation are generally attributed to trauma and deficiencies in the locking mechanism [11]. The method of fixation between the tibial component and PE insert differs among products. Locking mechanisms include peripheral capture, snap fit, dovetail, tongue-in-groove, and anti-rotational island, among others [5,6,16]. Manufacturers design these mechanisms with varying configurations, degrees, and positions, often combining multiple features.

Most previous studies comparing fixation methods and outcomes have been retrieval-based, focusing on backside wear by analyzing the location and extent of damage on the insert [4,5,6]. However, experimental studies that compare the stability of fixation based on different locking mechanisms—factors believed to be associated with PE dissociation—have yet to be reported. Therefore, this study evaluates the stability of fixation using actual implants with different locking mechanisms through validated experimental methods. We hypothesize that the force required for disassembly and reassembly varies depending on the type of locking mechanism.

## 2. Materials and Methods

This study conducted a disassembly test of the tibial inserts from three types of total knee arthroplasty implants following the ASTM F1814 standard [17]. Additionally, an assembly test of the tibial insert into the tibial plate was performed.

### 2.1. Total Knee Arthroplasty Implant Models

**Figure 1:**
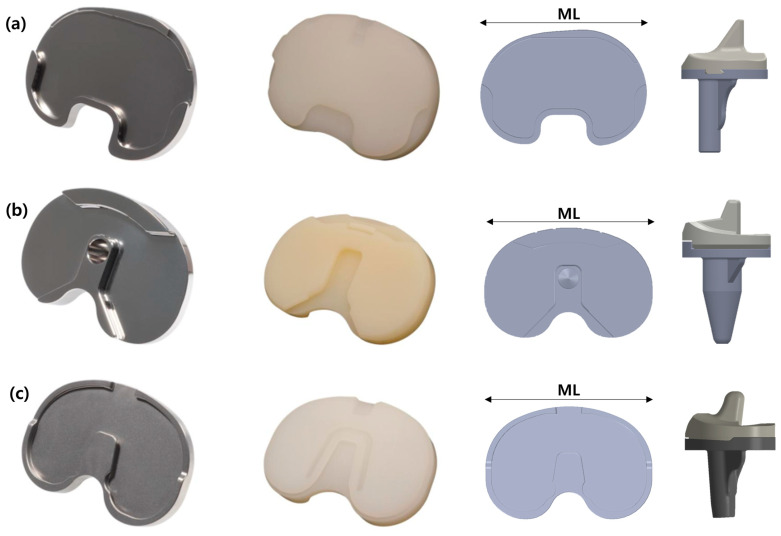
Representative photographs of the test specimens: (**a**) Group 1 (Anthem TKR, Smith & Nephew Inc., London, UK); (**b**) Group 2 (Attune™ TKR, DePuy Synthes, Warsaw, IL, USA); (**c**) Group 3 (PNK Knee, Skyve Co., Ltd., Seoul, Republic of Korea).

Three types of total knee arthroplasty implants (Groups 1, 2, and 3) were selected for the study. Each model represents a distinct design approach and material composition commonly used in clinical practice. Three tibial inserts from each model were prepared for testing. These models were commercially available products, and their details are shown in Figure 1. Each model was chosen based on its prevalence in clinical use and its unique design features. In Group 1, dovetail lips were located on the posterior, medial, and lateral sides 11. In Group 2, peripheral rims were present on both the anterior and posterior sides, along with a central anti-rotation island. Additionally, snap-fit structures surrounded the anterior peripheral rims of the insert, as shown in Figure 1b [18,19]. In Group 3, the tibial insert featured both a central anti-rotation island with a dovetail structure and a posterior-side peripheral rim. Snap-fit structures surrounded a female section of the dovetail locking mechanism on the backside of the insert, as depicted in Figure 1c.

### 2.2. Experimental Equipment

Universal Testing Machine (UTM): A UTM frame Type 5569A (Instron, Norwood, MA, USA) was used to perform the disassembly tests. This machine can control and measure both the force applied and the displacement of the test specimen with high accuracy.

Load Cell: A load cell with a capacity of ±50 kN was used to measure the force required to disassemble the tibial inserts. A load cell of type 2525-802 (Instron, Norwood, MA, USA) was selected for its high sensitivity and accuracy in force measurement, which is essential for detecting subtle differences between the models.

Displacement Transducer: A displacement transducer Type 5569A with a maximum range of 1212 mm (Instron, Norwood, MA, USA) was employed to measure displacement during the disassembly process. This sensor provided real-time data on the movement of the tibial inserts, enabling precise monitoring and recording of the disassembly process.

Room Climate Sensor: A Humlog 20 THIP (E+E Elektronik, Engerwitzdorf, Austria) was used as the room climate sensor in this test to monitor environmental conditions. 

### 2.3. Specimen Preparation

The tibial inserts from each knee prosthesis model (Groups 1, 2, and 3) were separated and prepared as test specimens. Groups 1, 2, and 3 were selected with similar sizes in terms of ML (medial–lateral) dimension. Group 1 used tibial component size 3, Group 2 used size 4, and Group 3 used size D, with all specimens having a consistent ML dimension of 68 mm. Each tibial insert was thoroughly cleaned using a standardized protocol to eliminate any contaminants or residues from the manufacturing process. After cleaning, the specimens were dried and inspected for defects or irregularities that could influence the test results. Only specimens that passed this inspection were included in the experiment. For each group (Groups 1, 2, and 3), three tibia insert specimens per each test were prepared to ensure reliable and consistent results. Additionally, for each group (Groups 1, 2, and 3), a single tibial component specimen was prepared for each test, as the tibial components possess significantly greater strength and stiffness compared to the tibial insert components.

### 2.4. Equipment Setup for Disassembly Test

The UTM was equipped with a calibrated load cell and displacement sensor. A custom-made fixture was then installed on the UTM to securely hold the tibial inserts and knee prosthesis components. Each tibial insert was carefully positioned in the fixture to ensure consistent orientation and alignment across all tests. Special attention was given to minimizing any initial preload or misalignment that could distort the results.

For the test series, one tibial baseplate per implant type was embedded using a sand-filled epoxy resin (RenLam^®^ CY219 and Ren^®^ HY 5162-1, Huntsman A.M., The Woodlands, TX, USA) and reused unless damage was observed. A new insert was used for each test. The tibial baseplates and corresponding tibial inserts were assembled manually following the manufacturer’s instructions.

**Figure 2:**
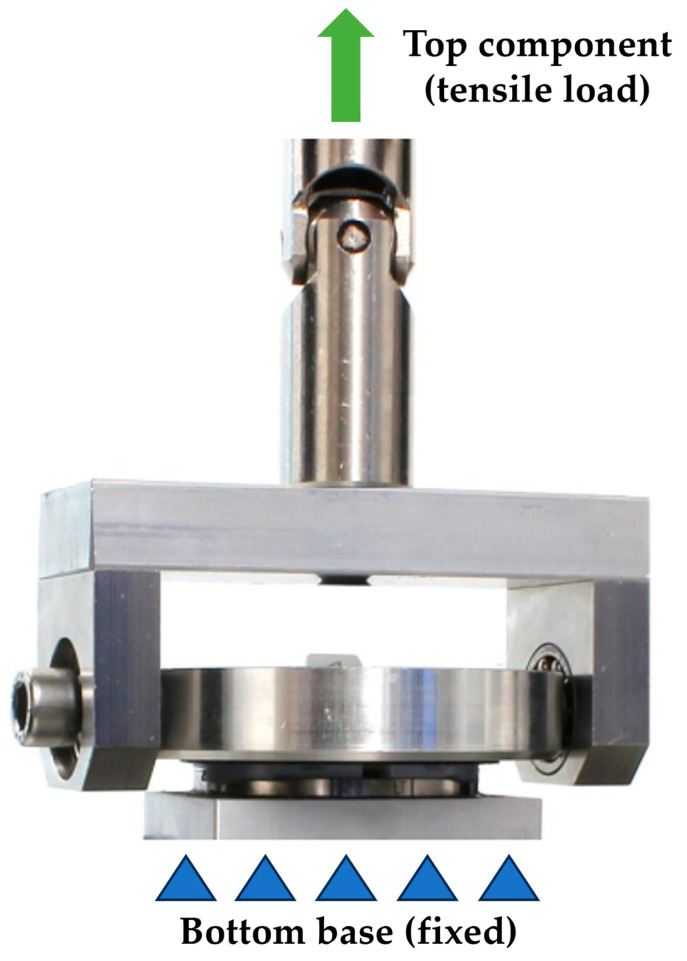
The setup for the static disassembly test in the tensile loading direction.

During the static axial tensile test, the tibial inserts were loaded in tensile mode to determine the disassembly load, as shown in Figure 2. A bottom base was fixed, and a tensile load was applied on a top component. A custom-made fixture was designed and fabricated to securely hold the tibial inserts and knee prosthesis components during testing. The fixture was constructed to ensure the tibial inserts were positioned in a manner that closely simulated their orientation and constraints within an actual knee prosthesis.

The load was applied using a 3D-printed plastic negative that matched the articulating surface of the tibial insert. The negative was bonded to the tibial insert using a two-component PE/PP adhesive (technicoll^®^ 9410, Zorneding, Germany).

A tensile disassembly loading rate of 5 mm/min was used. All tests were performed at room temperature. The bounding surface between the tibial baseplate and the tibial insert was moistened with deionized water to minimize frictional effects. The parameters used for the static tensile test are shown in Table 1.

### 2.5. Disassembly Test Procedure

The UTM was programmed to apply a controlled displacement at a constant rate to the tibial insert until disassembly occurred. Force and displacement data were continuously recorded throughout the test. The rate of displacement was selected based on the ASTM F1814 standard [17].

Each tibial insert was tested under identical conditions, and the process was repeated three times for each model to ensure the reliability and reproducibility of the results. Between each test, the fixture and UTM were reset to their initial conditions to eliminate any potential residual effects from previous tests.

The maximum force and corresponding displacement at the point of disassembly were recorded for each test. The data were averaged to obtain a mean value for each model, and the standard deviation was calculated to evaluate variability in the measurement.

### 2.6. Assembly Test Procedure

**Figure 3:**
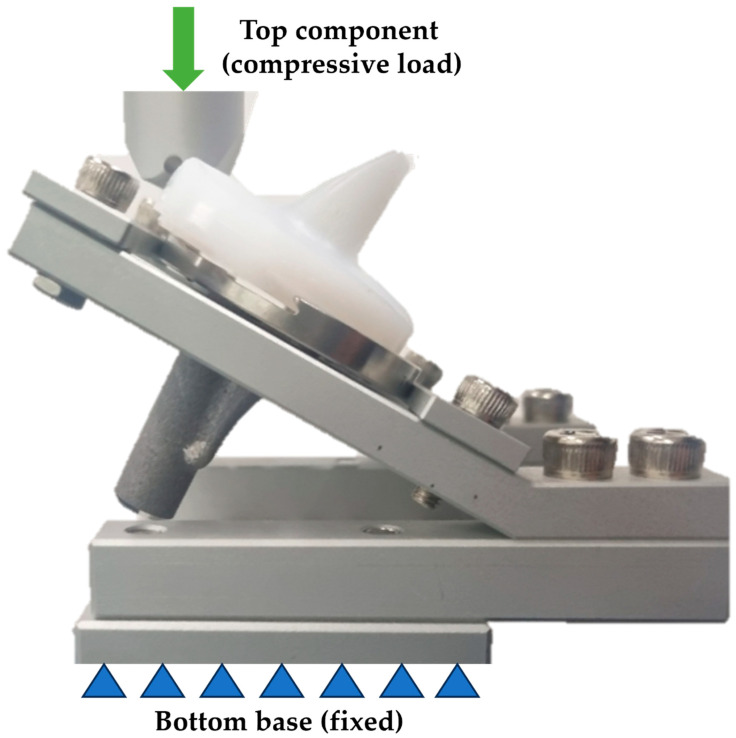
The setup for the static insertion test in the compressive loading direction.

The assembly of the tibial insert into the tibial plate was evaluated as follows. The UTM was programmed to apply a controlled force, with a maximum of 200 N, to the tibial insert until it was fully assembled with the tibial plate. A bottom base was fixed, and a compressive load was applied on a top component. Force and displacement data were continuously recorded throughout the test. Each tibial insert was tested under identical conditions, and the process was repeated three times for each model to ensure the reliability and reproducibility of the results. Between each test, the fixture and UTM were reset to their initial conditions to eliminate any potential residual effects from previous tests. The maximum force and corresponding displacement at the point of assembly were recorded for each test. The data were averaged to obtain a mean value for each model, and the standard deviation was calculated to assess variability in the measurements. A custom-made fixture was designed and fabricated to securely hold the tibial plate and prosthesis components during testing, as shown in Figure 3. The fixture was constructed to ensure the tibial plates were positioned in a manner that closely simulated their orientation and constraints within an actual knee prosthesis. The specimens were prepared by inserting the tibial insert into the tibial plate until it contacted the peripheral rim region.

## 3. Results

### 3.1. Disassembly Test of the Tibial Inserts

**Figure 4:**
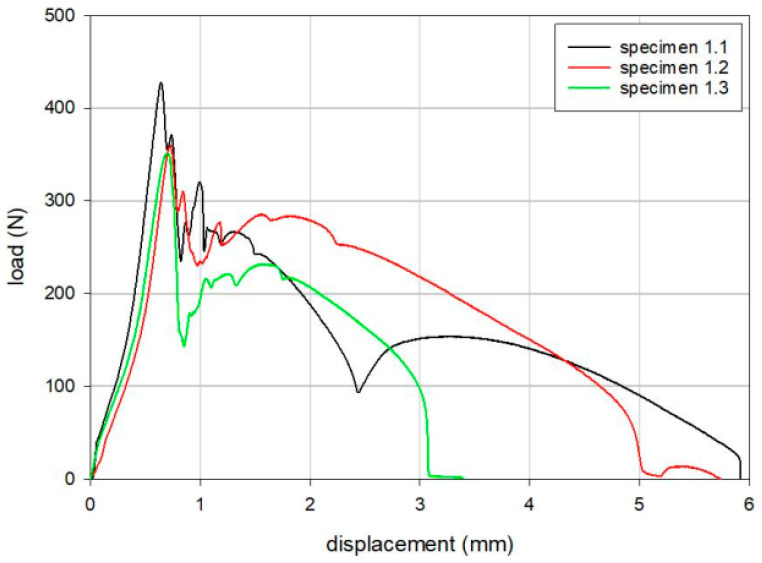
Load versus displacement curves for the tensile static disassembly test of Group 1 (Anthem TKR, Smith & Nephew Inc., London, UK).

**Figure 5:**
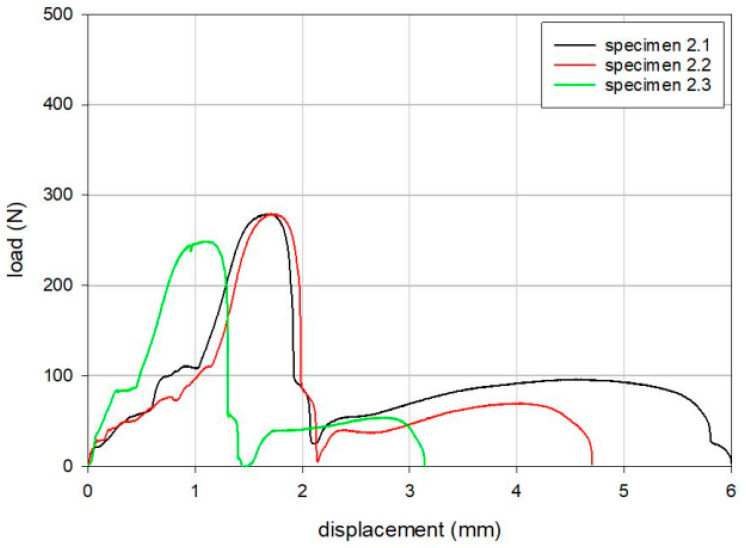
Load versus displacement curves for the tensile static disassembly test of Group 2 (Attune™ TKR, DePuy Synthes, Warsaw, IL, USA).

**Figure 6:**
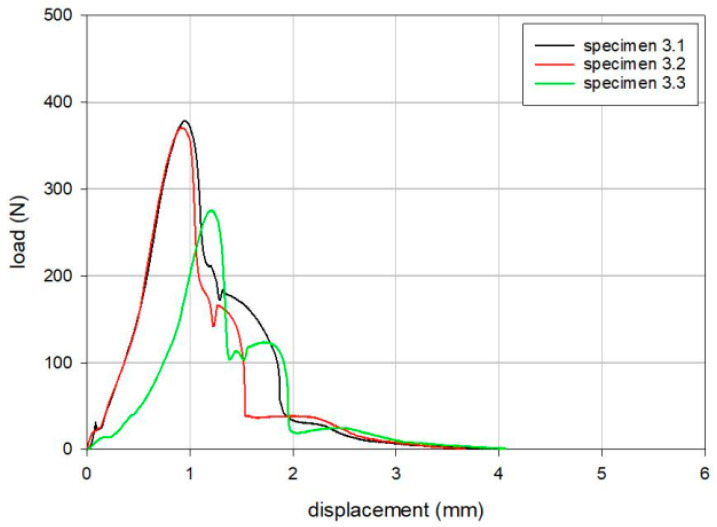
Load versus displacement curves for the tensile static disassembly test of Group 3 (PNK Knee, Skyve Co., Ltd., Seoul, Republic of Korea).

The results of the tensile loading tests are summarized in Table 2, Table 3 and Table 4. The maximum loads for Groups 1, 2, and 3 were 379 N, 269 N, and 342 N, respectively, following the order of Group 1, Group 3, and Group 2. Load versus displacement curves for the tensile static disassembly tests are shown in Figure 4, Figure 5 and Figure 6.

**Figure 7:**
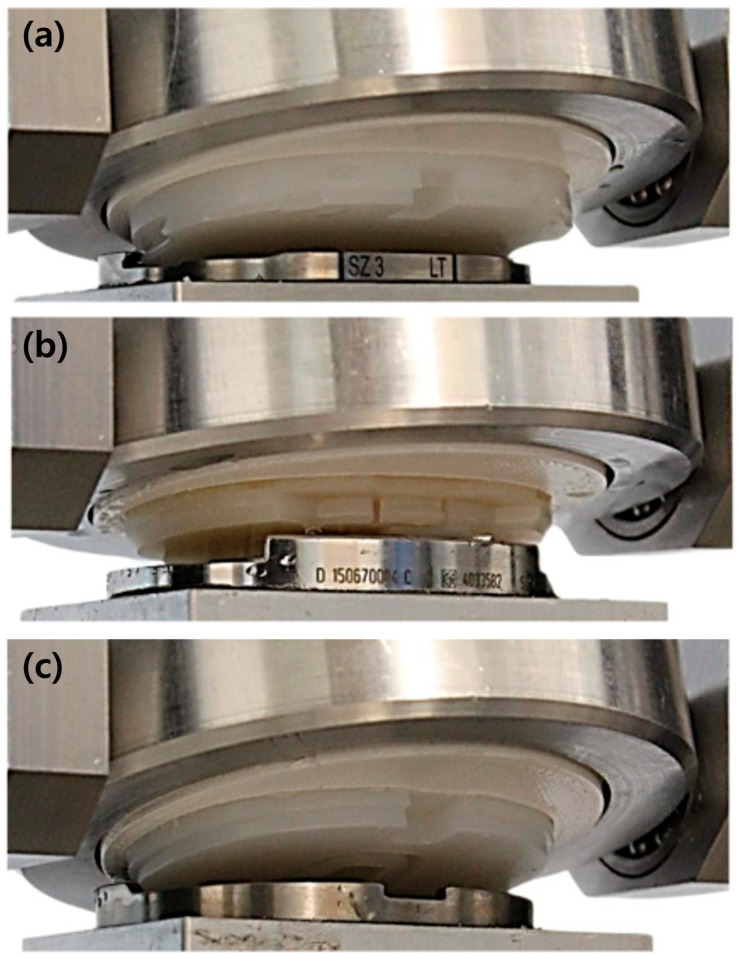
Representative photographs of the detached tibial insert due to the disassembly of the tibial insert from the tibial baseplate: (**a**) Group 1 (Anthem TKR, Smith & Nephew Inc., London, UK); (**b**) Group 2 (Attune™ TKR, DePuy Synthes, Warsaw, IL, USA); (**c**) Group 3 (PNK Knee, Skyve Co., Ltd., Seoul, Republic of Korea).

As a result of the tensile loading tests, all samples failed due to the disassembly of the tibial insert from the tibial baseplate, as shown in Figure 7.

### 3.2. Assembly Test of the Tibial Inserts

As a result of the assembly test, all tibial inserts and tibial baseplates were fully assembled. The results of the assembly test are shown in Table 5. The maximum loads for specimen assembly in Groups 1, 2, and 3 were 71 N, 49.7 N, and 48.7 N, respectively, following the order of Group 1, Group 2, and Group 3.

## 4. Discussion

The most important finding of this study is that Group 3, which features a snap-fit structure, required the lowest assembly force and demonstrated a moderate disassembly force. The force required for assembly between the tibial component and PE insert does not necessarily correlate with the force required for disassembly and appears to vary depending on the type of locking mechanism.

There are considerations to take into account before interpreting these results. This study was conducted using new, unused products, which inherently limited the sample size. Consequently, any outliers in the data could significantly influence the results. To address this, we assumed that values showing unusually large deviations compared to others were outliers. This applied to the first disassembly test in Group 1 and the third disassembly test in Group 3. While a larger sample size would allow for more precise analysis, we believe that our findings hold sufficient clinical implications within the realistic constraints of the study.

Among the reported literature, some cases of dissociation were caused by trauma [14,15,20,21], while others suggested that anterior lift-off led to dissociation [22]. Therefore, strong fixation is considered an essential requirement to prevent dissociation. In our tensile static disassembly test, Group 1 demonstrated the highest strength, which was attributed to the large contact area of the dovetail structures located on the posterior, medial, and lateral sides. Excluding outliers, Group 3 exhibited high strength comparable to Group 1. The greater strength observed in Groups 1 and 3 compared to Group 2 was due to the presence of the dovetail structure in both the insert and the tibial plate [18]. Group 3, which incorporates both a dovetail structure and a peripheral rim, exhibited 27% higher strength than Group 2.

In the assembly test, compared to the insert in Group 1, the snap-fit structures around the anterior peripheral rims in Group 2 and the female section of the dovetail locking mechanism on the backside of the insert in Group 3 enabled smoother insertion of the tibial insert into the tibial plate. PE insert dissociation is an uncommon complication. However, a significant number of cases reported in the literature involved the Group 1 product used in our study [9,11,12,13,14]. In our study, Group 1 demonstrated considerable fixation strength but also required a significantly higher force for assembly. This indicates that achieving complete fixation is relatively challenging, which may explain the higher number of dissociation cases reported with this design. In et al. suggested that the high incidence of dissociation with the Group 1 product was due not only to the locking mechanism but also to limited visibility caused by the authors’ mini-subvastus approach [11]. The Group 1 design requires a substantial amount of force for secure fixation, which may increase the likelihood of incomplete fixation. Additionally, incomplete fixation could be difficult to detect due to the limited visibility associated with certain surgical techniques. Rutten et al. reported a case where a posterior femoral osteophyte caused impingement, leading to late dissociation [9]. However, this may also have been due to interference with achieving complete fixation. By contrast, Groups 2 and 3 required relatively less force for assembly, making complete fixation more likely to be achieved. This reduction in assembly force may decrease the likelihood of PE insert dissociation caused by initial fixation failure.

Excessive force required to achieve secure fixation may lead to additional negative effects. Since most patients undergoing TKA are elderly and likely to have osteoporosis, intraoperative tibial fractures can occur, particularly during final cementation or implantation [23,24]. Most implant systems require the use of an impactor to insert PE insert by striking it into place. If the assembly force required for the insert is high, it may increase the risk of such fractures. Therefore, inserts that are easier to assemble could potentially reduce the risk of these complications.

An interesting finding is that Group 3, which demonstrated an appropriate disassembly force, exhibited the lowest assembly force. This outcome can be attributed to the snap-fit structures present in the tibial insert [25]. Additionally, the dovetail mechanism applied to the posterior region exhibited strong disassembly force. Previous studies have shown that the dovetail locking mechanism preserves structural integrity even under loads significantly exceeding normal levels, while also reducing micromotion and the associated risk of osteolysis [11].

Previous studies have reported that implants utilizing a dovetail locking mechanism exhibited a higher degree of micromotion compared to those with peripheral rim mechanisms [16]. These studies also demonstrated that the central island in certain knee designs effectively reduces micromotion [16]. Group 3 features a design that integrates all of these advantages. The anterior region utilizes a peripheral rim mechanism, while the dovetail mechanism includes a significantly larger central island compared to the smaller structures in Group 1. The characteristics of this design have been validated by the performance of Group 3 in our study.

The present study has two main limitations. First, regarding our study design, the sample size for implants in each group was relatively limited. To address this limitation, experiments were conducted using implants of the most similar sizes and thicknesses for each group. In fact, retrieval-based studies cannot control for implant size and thickness, which are known to affect fixation strength. Fixation strength varies depending on the size of the tibial insert and tibial component. Likewise, previous studies have not demonstrated consistent effects of polyethylene size and thickness on backside wear and damage [26]. Second, as this is an experimental study, long-term results are needed to establish its clinical relevance.

Despite these limitations, this study conducted disassembly and assembly tests of tibial inserts from three types of knee prostheses following the ASTM F1814 standard [17]. By comparing the performance of each model, valuable insights were gained into the design and structural integrity of these prostheses. The results of this study provide essential information that can be used to improve the design and performance of knee prostheses, ultimately contributing to better clinical outcomes for patients.

## 5. Conclusions

Due to its snap-fit structure, Group 3 exhibited the lowest assembly force while utilizing the dovetail mechanism to demonstrate strong disassembly force. The detailed analysis and robust methodology employed in this study ensure that the findings are reliable and can serve as a reference for future research and development in this field.

## Figures and Tables

**Table 1:** Test parameters used for the static tensile test.

Parameter	ASTM F1814 [17]
Tensile loading rate	5 mm/min
Load application	Double cardan joint
Load transfer	Glued negative matching the articulating surface
Test environment	Bounding surface moistened with deionized water at room temperature

**Table 2:** Results of the static tensile disassembly test for Group 1 (Anthem TKR, Smith & Nephew Inc., London, UK).

Specimen	Maximum Load (N)	Displacement at Maximum Load (mm)
1.1	428	0.6
1.2	360	0.7
1.3	351	0.7
Mean	379	0.7
SD	42	0.0

**Table 3:** Results of the static tensile disassembly test for Group 2 (Attune™ TKR, DePuy Synthes, Warsaw, IL, USA).

Specimen	Maximum Load (N)	Displacement at Maximum Load (mm)
2.1	279	1.7
2.2	279	1.7
2.3	249	1.1
Mean	269	1.5
SD	18	0.3

**Table 4:** Results of the static tensile disassembly test for Group 3 (PNK Knee, Skyve Co., Ltd., Seoul, Republic of Korea).

Specimen	Maximum Load (N)	Displacement at Maximum Load (mm)
3.1	379	1.0
3.2	371	0.9
3.3	276	1.2
Mean	342	1.0
SD	58	0.2

**Table 5:** Results of the assembly test for Group 1 (Anthem TKR, Smith & Nephew Inc., London, UK); Group 2 (Attune™ TKR, DePuy Synthes, Warsaw, IL, USA); and Group 3 (PNK Knee, Skyve Co., Ltd., Seoul, Republic of Korea).

Specimen	Maximum Load (N)		
	Group 1	Group 2	Group 3
#1	71	50	51
#2	74	48	43
#3	68	51	51
Mean	71	49.7	48.7
SD	3	1.5	2.1

## Data Availability

Data are contained within the article.

## Abbreviations

The following abbreviations are used in this manuscript:

FB	Fixed-bearing
TKA	Total knee arthroplasty
PE	Polyethylene
UTM	Universal testing machine

## References

[1] Capella M., Dolfin M., Saccia F. (2016). Mobile bearing and fixed bearing total knee arthroplasty. Ann. Transl. Med..

[2] Engh G.A., Lounici S., Rao A.R., Collier M.B. (2001). In vivo deterioration of tibial baseplate locking mechanisms in contemporary modular total knee components. J. Bone Jt. Surg. Am..

[3] Diamond O.J., Doran E., Beverland D.E. (2018). Spinout/dislocation in mobile-bearing total knee arthroplasty: A report of 26 cases. J. Arthroplast..

[4] Levine R.A., Lewicki K.A., Currier J.H., Mayor M.B., Van Citters D.W. (2016). Contribution of micro-motion to backside wear in a fixed bearing total knee arthroplasty. J. Orthop. Res..

[5] Łapaj Ł., Mróz A., Kokoszka P., Markuszewski J., Wendland J., Helak-Łapaj C., Kruczyński J. (2017). Peripheral snap-fit locking mechanisms and smooth surface finish of tibial trays reduce backside wear in fixed-bearing total knee arthroplasty: A retrieval analysis of 102 inlays. Acta Orthop..

[6] Sisko Z.W., Teeter M.G., Lanting B.A., Howard J.L., McCalden R.W., Naudie D.D., MacDonald S.J., Vasarhelyi E.M. (2017). Current total knee designs: Does baseplate roughness or locking mechanism design affect polyethylene backside wear?. Clin. Orthop. Relat. Res..

[7] Gallo J., Goodman S.B., Konttinen Y.T., Wimmer M.A., Holinka M. (2013). Osteolysis around total knee arthroplasty: A review of pathogenetic mechanisms. Acta Biomater..

[8] Ries M.D. (2004). Dissociation of an ultra-high molecular weight polyethylene insert from the tibial baseplate after total knee arthroplasty: A case report. J. Bone Jt. Surg. Am..

[9] Rutten S., Janssen R. (2009). Spontaneous late dislocation of the high flexion tibial insert after Genesis II total knee arthroplasty. A case report. Knee.

[10] Chen C.-E., Juhn R.-J., Ko J.-Y. (2011). Dissociation of polyethylene insert from the tibial baseplate following revision total knee arthroplasty. J. Arthroplast..

[11] In Y., Sur Y.-J., Won H.-Y., Moon Y.-S. (2011). Recurrent dissociation of the tibial insert after mini-subvastus posterior-stabilized total knee arthroplasty: A case report. Knee.

[12] Lee D.-H., Lee T.G., Park S.-J., Han S.-B. (2013). Spontaneous late dissociation of the tibial insert after high-flex posterior-stabilized Genesis II total knee arthroplasty. J. Arthroplast..

[13] Voskuijl T., Nijenhuis T.A., Van Hellemondt G.G., Goosen J. (2015). Insert dissociation after fixed bearing PS constrained Genesis II total knee arthroplasty. A case series of nine patients. Acta Orthop. Belg..

[14] Agarwala S., Vijayvargiya M. (2018). Traumatic dissociation of the tibial insert with patellar tendon rupture after high-flex posterior-stabilized Genesis II total knee arthroplasty. Rev. Bras. Ortop..

[15] Reddy A.G., Rajan D.S., Chiranjeevi T., Karthik C., Kiran E.K. (2016). Failure of polyethelene insert locking mechanism after a posterior stabilised total knee arthroplasty-a case report. J. Orthop. Case Rep..

[16] Bhimji S., Wang A., Schmalzried T. (2010). Tibial insert micromotion of various total knee arthroplasty devices. J. Knee Surg..

[17] (2022). Standard Guide for Evaluating Modular Hip and Knee Joint Components.

[18] Triathlon® Total Knee System Design Rationale. STRYKER. TRIATH-BRO-12_Rev-1_25139. https://www.stryker.com/content/dam/stryker/no-index/training-and-education/jr45/june/resources/Triathlon%20Design%20Rationale.pdf.

[19] (2013). Value Brief: Attune Knee System.

[20] Hedlundh U., Andersson M., Enskog L., Gedin P. (2000). Traumatic late dissociation of the polyethylene articulating surface in a total knee arthroplasty—A case report. Acta Orthop. Scand..

[21] Anderson J.A., MacDessi S.J., Della Valle A.G. (2007). Spontaneous, recurrent dislodgment of the polyethylene tibial insert after total knee arthroplasty: A case report. J. Bone Jt. Surg. Am..

[22] Davis P.F., Bocell J.R., Tullos H.S. (1991). Dissociation of the tibial component in total knee replacements. Clin. Orthop. Relat. Res..

[23] Agarwala S., Bajwa S., Vijayvargiya M. (2019). Intra-operative fractures in primary total knee arthroplasty. J. Clin. Orthop. Trauma.

[24] Seidman A., Green A., McCall D., Finch J., Smith L.C. (2021). Intraoperative tibia fractures during primary total knee arthroplasty. Cureus.

[25] Klahn C., Singer D., Meboldt M. (2016). Design guidelines for additive manufactured snap-fit joints. Procedia CIRP.

[26] Conditt M.A., Thompson M.T., Usrey M.M., Ismaily S.K., Noble P.C. (2005). Backside wear of polyethylene tibial inserts: Mechanism and magnitude of material loss. J. Bone Jt. Surg. Am..

